# Fluorescence Reduction Neutralization Test: A Novel, Rapid, and Efficient Method for Characterizing the Neutralizing Activity of Antibodies Against Dengue Virus

**DOI:** 10.3390/cimb47030140

**Published:** 2025-02-21

**Authors:** Jiazheng Guo, Jiansheng Lu, Peng Du, Kexuan Cheng, Chao Lei, Yujia Jiang, Meiling Peng, Yating Li, Kaiyue Sun, Changyan Xu, Yunzhou Yu, Chen Gao, Qinglin Kang, Yixiao Zhang, Rong Wang, Zhixin Yang

**Affiliations:** Laboratory of Advanced Biotechnology, Beijing Institute of Biotechnology, Beijing 100071, China; sdqzgjz@163.com (J.G.); lujiansheng2008@163.com (J.L.); dudedu@sina.com (P.D.); 16696653458@163.com (K.C.); lei2272830754@163.com (C.L.); jiangyujia19990627@163.com (Y.J.); pml517@163.com (M.P.); lyt18214729367@163.com (Y.L.); sunkaiyue1999@outlook.com (K.S.); xcy3146393080@163.com (C.X.); yunzhouyu@163.com (Y.Y.); chengao2015@163.com (C.G.); kql_lynn@163.com (Q.K.); 18253663290@163.com (Y.Z.)

**Keywords:** DENV, antibody, rapid screening platform, neutralization activity assay, FRNT_50_

## Abstract

Dengue virus (DENV) is a major public health threat in the tropical and subtropical regions of the world. Climate change resulting from global warming is further expanding DENV–endemic areas, adversely affecting public life and health. Despite this, no specific drug against DENV has been developed so far. Vaccines and neutralizing antibodies are the chief preventive and therapeutic tools for managing pathogenic infections. The present study describes the development of a novel fluorescence reduction neutralization test (FRNT) for evaluating the neutralizing activity of antibodies against DENV. This FRNT allows rapid antibody screening. In addition, we calculated the FRNT_50_ to indicate the neutralizing ability of the antibodies. In contrast to the conventional plaque reduction neutralization assay, the FRNT has a shorter experimental cycle, a simpler operation, and greater objectivity, which can greatly accelerate the research and development process of vaccines and antibodies against DENV.

## 1. Introduction

Dengue virus (DENV), a member of the *Flaviviridae* family, is a positive-sense single-stranded RNA (+ssRNA) virus. It contains three structural and seven non-structural proteins. DENV is commonly classified into four serotypes (DENV I–IV) based on antigenic differences. There are reports that DENV-V has been found in Malaysia, but the serotype is currently being verified [1]. It is mainly transmitted by *Aedes aegypti* and *Aedes albopictus*. Increasing global warming in recent years has expanded the epidemic area for dengue worldwide, putting approximately 3.6 billion people at risk of infection [2], and posing a grave threat to their lives and property. Antibody-dependent enhancement (ADE) is crucial for the pathogenesis of patients severely affected by dengue. When people first acquire the DENV infection, they develop mild symptoms because the virus stimulates good cellular and humoral immunity, providing lifelong immunity against the DENV subtype. However, when people get re-infected with other DENV subtypes, the antibodies produced during the first infection direct the virus to attack its target cells, leading to a massive proliferation of DENV and causing severe dengue hemorrhagic fever, dengue shock syndrome, and even death [3]. Antigen detection and nucleic acid testing are commonly utilized for the diagnosis of dengue virus infection. However, due to the potential cross-reactivity of serological tests, they have been gradually replaced by nucleic acid testing in China. The development of a dengue vaccine is a highly challenging task. Many pharmaceutical companies and academic institutions, including Sanofi Pasteur and Takeda Pharmaceutical, are developing safe and effective vaccines that can be used against all four serotypes of dengue [4]. No vaccine against dengue has yet been developed that can be used in all age groups [3].

Neutralizing antibody is an important tool in the treatment of pathogenic infections [5,6]. These antibodies prove effective in preventing DENV infections [7,8,9]. The plaque reduction neutralization test (PRNT), developed by Russel and Nisalak in 1967 [10], is regarded as the gold standard for testing antibody neutralization potency [11,12]. However, PRNT requires an experimental period of 4–6 days and a manual counting of the number of plaques formed in the cell culture plate, making it a time-consuming and subjective method, resulting in large variations between sample wells. Some institutions have optimized the PRNT by improving the culture conditions or the type of medium, and have successfully shortened the experimental period and simplified the experimental manipulations [13]. Other similar assays have also been developed, such as the focus reduction neutralization test, which calculates antibody neutralization potency based on the reduction in the number of foci of infected cells [14]. However, similar to the PRNT, the focus reduction neutralization test is also a count-based assay with high variability. In the commonly used 24-well plate, it can only have one duplicate sample per well plate. All the aforementioned methods are time-consuming and cumbersome, and are hence unsuitable for the large-scale evaluation of vaccines and antibodies. Therefore, it is essential to improve the current common gold standard assay to increase assay throughput and reduce experimental time.

Here, we established a novel method for evaluating antibodies. This method allows direct assessment of the degree of viral infection in host cells based on fluorescence intensity at specific wavelengths, which correlates to the neutralization potency of the antibody, and allows calculation of the neutralizing capacity of the antibody.

## 2. Materials and Methods

### 2.1. Virus Cultures and Cell Lines

DENV I strain GE27, DENV II strain New Guinea C (NGC), DENV III strain YN01, and DENV IV strain 30 were stored at Beijing Institute of Biotechnology. All viruses were cultured and produced in C6/36 cells (ATCC, Manassas, VA, USA). Culture supernatants from the virus-infected cells were centrifuged at 4000× *g* to remove cellular debris. The viruses were then dispensed into cryopreservation tubes and stored at −80 °C.

The C6/36 cells were cultured in Roswell Park Memorial Institute (RPMI)-1640 medium with 10% fetal bovine serum (FBS; Excel, New Taipei City, Taiwan). BHK-21 (ATCC) cells were cultured in Dulbecco’s modified Eagle medium (DMEM) medium with 10% FBS serum. FreeStyle™ 293-F cells used for antibody expression were cultured in FreeStyle 293 Expression Medium 12338 (Gibco, Grand Island, NY, USA). C6/36 cells were cultured at 28 °C without CO_2_. For producing DENV, C6/36 cells were cultured in the same way, except for FBS, which was adjusted to 2%. BHK-21 cells were cultured at 37 °C with 5% CO_2_.

### 2.2. Viral Titer Assay: Plaque Test

BHK-21 cells (8 × 10^5^ cells) were seeded into a 6-well plate (2 mL cells per well) and incubated at 37 °C with 5% CO_2_ for 24 h. Next, DENV was added to these 6-well plates through a 2-fold gradient dilution of DMEM and incubated at 37 °C for 1 h. The BHK-21 cells were washed two times with sterile PBS to remove invalid viruses. The incubation supernatant was discarded. A semi-solid medium at room temperature, consisting of 2× DMEM containing 4% (*v*/*v*) FBS and 2% (*w*/*v*) low-melting-point agarose (0815, Amresco, Solon, OH) in a ratio of 1:1, was added to the incubated 6-well plates (2 mL per well). After solidification of the semi-solid medium, the 6-well plates were incubated at 37 °C under 5% CO_2_ for 4–5 days until a plaque formed.

### 2.3. Viral Titer Assay—Fluorescence Test

BHK-21 cells (4 × 10^5^ cells/mL) were seeded into 96-well cell culture plates (100 μL cells per well) and cultured at 37 °C under 5% CO_2_ for 24 h. DENV was added to the 96-well plates (50 μL per well) through a 2-fold gradient dilution in DMEM and incubated at 37 °C for 1–2 h. After discarding the incubation supernatant, the 96-well cell culture plate was washed twice with sterile PBS. Then, DMEM containing different FBS concentrations (150 μL per well) was added to aforementioned 96-well cell plates and incubated at 37 °C under 5% CO_2_ for 24–72 h. Thereafter, the BHK-21 cells were fixed and perforated by 4% (*v*/*v*) paraformaldehyde (with 0.2%, *v*/*v*, Triton X-100) at 4 °C for 30 min. To block non-specific binding, 2% bovine serum albumin (BSA) (100 μL per well) was used to incubate the plates for 2 h at room temperature. Subsequently, the detection antibody 4G2 (AB41349, abcam) was used to incubate the cell plate (100 μL per well) overnight at 4 °C. Afterward, the cell plates were washed with PBS five times for 3 min each time. Then, a mixture of goat polyclonal antibody (pAb) and mouse (Ms) IgG conjugated with Alexa Fluor 488 (*v*/*v* 1:3000; AB150113, abcam) was used to incubate the cell plates for 1 h in the dark at room temperature. At the end of the incubation, the plates were again washed five times with PBS for 3 min each time. The results were obtained by detecting reflected light at a wavelength of 535 nm using a SpectraMax i3x (Molecular Devices, San Jose, CA, USA).

### 2.4. Plaque Reduction Neutralization Test (PRNT)

A heavy chain antibody 2E4-3G against dengue virus E protein was obtained early in the laboratory by phage display technology. This antibody was selected as a sample for validation of neutralizing activity in this study. BHK-21 cells (8 × 10^5^ cells) were seeded into 6-well plates (2 mL per well) and incubated at 37 °C under 5% CO_2_ for 24 h. The antibodies were diluted 3-fold using DMEM, added to approximately 100 pfu of DENV IV, and incubated at 4 °C for 1 h. The resulting antibody–virus suspension was added to a 6-well plate cultured with BHK-21 cells and incubated at 37 °C for 1 h. After discarding the supernatant, the obtained semi-solid medium at room temperature, comprising a mixture of 2× DMEM with 4% (*v*/*v*) FBS and 2% (*w*/*v*) low-melting-point agarose in a ratio of 1:1, was added to the incubated 6-well plates (2 mL per well). After solidifying the medium, the 6-well plates were incubated at 37 °C under 5% CO_2_ for 4–5 days until a plaque formed. The antibody concentration responsible for a 50% reduction in plaque, termed PRNT_50_, was derived from a nonlinear curve fit in GraphPad Prism software 8.0 (GraphPad Inc., La Jolla, CA, USA).

### 2.5. Fluorescence Reduction Neutralization Test (FRNT)

BHK-21 cells (4 × 10^5^ cells/mL) were seeded into 96-well cell culture plates (100 μL per well) and cultured at 37 °C with 5% CO_2_ for 24 h. The antibody was diluted 3-fold by DMEM and added to DENV. The resulting antibody–virus supernatant was immediately added to BHK-21 cells and incubated at 37 °C for 1 h. After discarding the supernatant, the BHK-21 cells were washed three times with sterile PBS. DMEM was added to the cells and incubated for 48 h at 37 °C under 5% CO_2_. Thereafter, BHK-21 cells were fixed and perforated using 4% (*v*/*v*) paraformaldehyde (with 0.2%, *v*/*v*, Triton X-100) at 4 °C for 30 min. To block non-specific binding, 2% BSA was used to incubate the plates (100 μL per well) for 2 h at room temperature. Detection antibody 4G2 was used to incubate the cell plate (100 μL per well) overnight at 4 °C. Afterward, the cell plates were washed with PBS five times for 3 min each time. Then, goat pAb to Ms IgG Alexa Fluor 488 (*v*/*v*, 1:3000) was used to incubate the cell plates for 1 h in the dark at room temperature. The plates were similarly washed five times with PBS (3 min each time) at the end of the incubation. The results were obtained by detecting reflected light at a wavelength of 535 nm using SpectraMax i3x (Molecular Devices, San Jose, CA, USA). The antibody concentration that causes a reduction of 50% in fluorescence intensity was termed FRNT_50_ and calculated through nonlinear curve fitting using GraphPad Prism 8.0.

## 3. Results

### 3.1. Production and Detection of DENV

**Figure 1:**
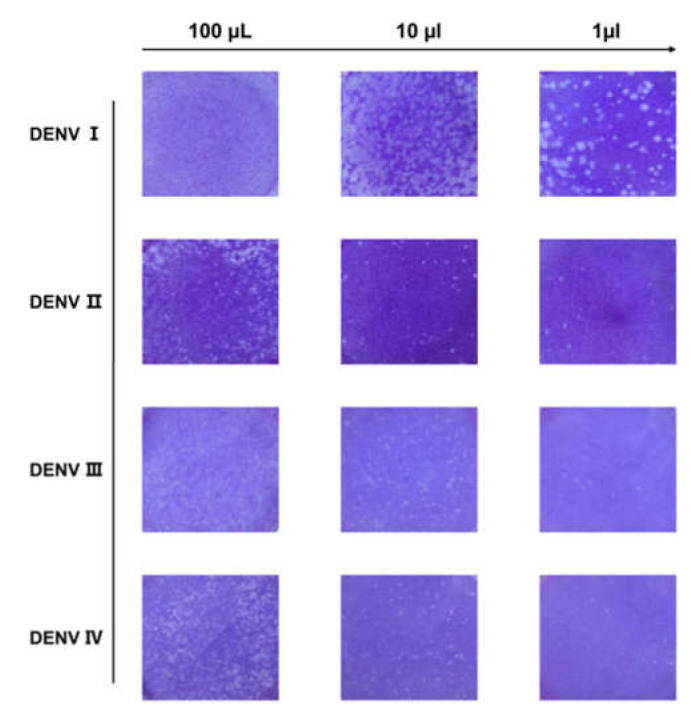
Plaque reduction test for dengue virus (DENV). Schematic representation of a plaque with four serotypes of DENV corresponding to 100, 10, and 1 μL of dengue virus, respectively. As the virus is diluted, plaques showing a decreasing gradient can be observed. Viral titer was calculated from the count of plaques.

DENV I–IV produced by C6/36 cells were first identified through the plaque reduction assay. From its initial volume of 100 μL, the DENV wase serially diluted to 10-fold and subsequently added to a six-well plate cultured with BHK-21 cells for plaque formation. As shown in Figure 1, a series of plaques formed, which showed a clear gradient decrease with increasing volume dilution. The number of plaques was counted to determine the DENV titer. As shown in Table 1, the titers of DENV I–IV were all above 10^4^ pfu/mL and could be used for subsequent studies.

### 3.2. Construction and Optimization of the FRNT for DENV

**Figure 2:**
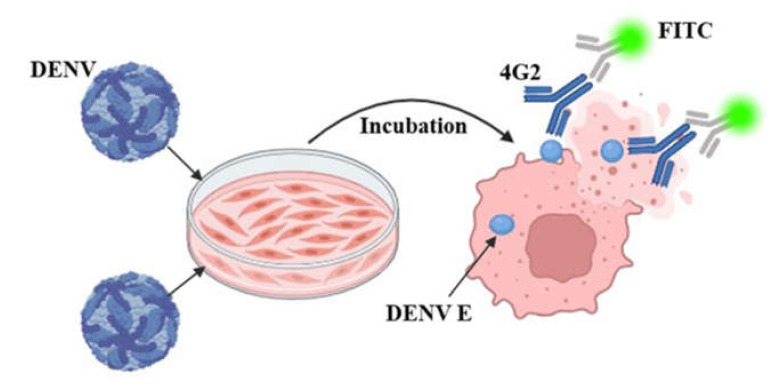
Principles of fluorescence reduction analysis methods.

To identify DENV titers more rapidly and accelerate the development of vaccines and neutralizing antibodies, we established a fluorescence intensity-based assay (Figure 2). A previous study identified 4G2 as a universal detection antibody for DENV I–IV with a binding site in the conserved region of protein E [15]; DENV titers are determined by the fluorescence intensity of the anti-4G2 antibody (Alexa Fluor 488,Abcam, Cambridge, UK). As shown in Table 2, different parameters, such as the incubation time, FBS concentration, and 96-well plate type, were used to explore the optimal experimental conditions.

**Figure 3:**
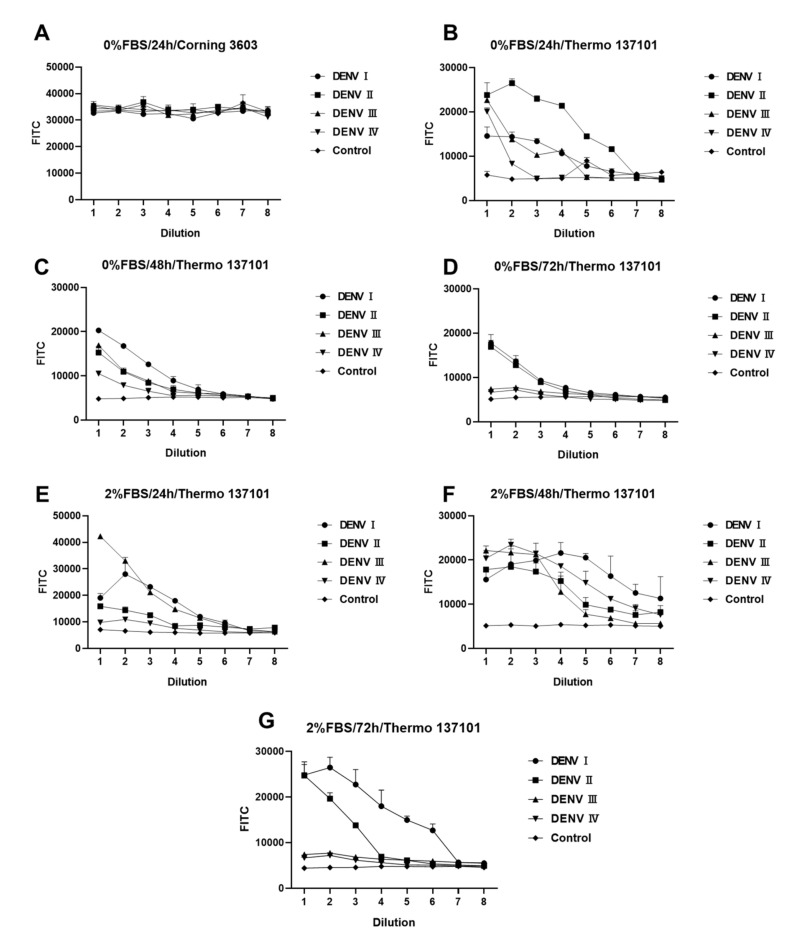
Fluorescence reduction analysis of dengue virus (DENV). (**A**–**G**) Fluorescence intensity analyses of DENV-infected BHK-21 cells under different conditions. Difference between (**A**,**B**) conditions for 96-well culture plates. (**B**–**D**) differs from (**E**–**G**) in fetal bovine serum (FBS) concentration. (**B**–**D**) and (**E**–**G**) differ in the duration of incubation after infection with DENV. All DENV samples were diluted in a 2-fold gradient beginning from 50 μL. Data are expressed as a multipoint mean relative fluorescence intensity (535 nm) ± standard deviation (SD) of two parallel wells in a 96-well plate (n = 2).

Figure 3 shows differences in DENV infection under different experimental conditions. Every experiment was repeated twice with two multiple wells (n = 2). Light reflected from the material of the 96-well cell culture plate itself has a large effect on the results (Figure 3A,B). Thermo 13701, with its black border and black background, is more suitable for experimental designs that need background values significantly lower than those of the sample well. The concentration of FBS is another chief factor that affects the experiment. As shown in Figure 3, some DENV serotypes need FBS during infection. Such serotypes grow better in DMEM containing 2% FBS. The incubation time of DENV in BHK-21 cells is another major factor. Figure 3E–G indicate a large variation in the amplification rate of different serotypes of DENV. After 24 h of infection, DENV I and DENV IV had higher titers, whereas DENV II and DENV III had lower titers. An increase in the titers of DENV II was noted, while DENV IV, which previously had higher titers, showed a substantial decrease. After 48 h of infection, the peak fluorescence intensity of DENV I–IV was approximately 2 × 10^4^, demonstrating a certain gradient with dilution. Based on the results obtained, the following factors were identified as the optimal conditions for the experimental design: 2% FBS, 48 h incubation, and a black background plate (Thermo 137101). Under the optimal experimental conditions (2% FBS and 48 h), the sample wells differed significantly from the control wells. The fluorescence intensity showed a gradient relationship with concentration. Hence, this design can be used for screening and evaluating antiserum or neutralizing antibodies for DENV.

### 3.3. Rapid Screening of Neutralizing Antibodies Against DENV Using the FRNT

**Figure 4:**
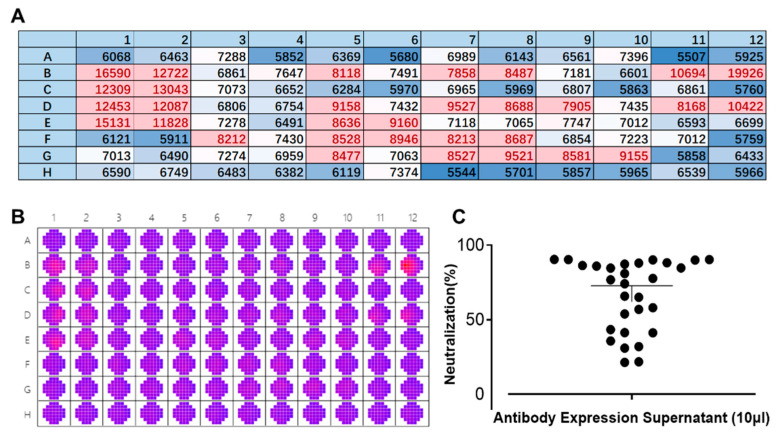
Rapid screening of neutralizing antibodies by the fluorescence reduction neutralization test (FRNT). (**A**) Heat map presentation of the scanned wells of a 96-well plate at 535 nm. Red: well is infected with a high titer of dengue virus (DENV); blue: well is infected with a low titer of DENV or background color. Each value/well is the average of 37 multipoints. Numbers and letters represent the layout of the 96-well plates. (**B**) Raw data of a 96-well plate after scanning its wells by a SpectraMax i3x at a wavelength of 535 nm. A11 and A12 are blank controls, and B11 and B12 are controls for DENV without antibody. Red: well is infected with a high titer of dengue virus (DENV); purple: well is infected with a low titer of DENV or background color. (**C**) A scatterplot showing the neutralization effects of different antibody supernatants against DENV IV.

We employed a laboratory-constructed antibody library in the early stages of our research. Multiple rounds of screening were carried out to identify the antibody sequences that can target the E protein. The newly designed FRNT was used instead of the conventional PRNT for evaluating antibody neutralizing activity. The supernatant of 293-F cells expressing antibodies was mixed with 25 µL/well of DENV IV (corresponding to approximately 425 pfu) and then used to infect BHK-21 cells for 1 h at 37 °C. The following steps were consistent with result 3.2. As shown in Figure 4B, significant differences in the fluorescence spectrum of DENV can be observed by wellscan. Multipoint means that each well was averaged to create a heat map (Figure 3A). A comparison of the colors clearly showed the strength of the neutralizing effect of the antibody against DENV. Transferring the data into a scatterplot (Figure 4C) enabled a more intuitive description of the inhibition rate of DENV against dengue. Based on the results obtained, it is evident that this method facilitates the rapid screening of neutralizing antibodies (within 3 days), which greatly accelerates the speed with which candidate antibodies are obtained. During this screening, twelve antibodies were obtained, with at least a 75% inhibition rate against DENV IV.

### 3.4. Validation of Antibody Neutralization Activity In Vitro

Half-neutralizing concentration is an important indicator in the evaluation of neutralizing antibodies. Previously, the PRNT_50_ obtained by PRNTs was the most used indicator of antibodies against DENV neutralization in vitro [7,16]. To verify whether our proposed FRNT method can be used to quantify the neutralizing effect of antibodies, we obtained an FRNT_50_ value similar to the PRNT_50_ to represent the neutralizing effect of antibodies. The FRNT and PRNT were designed to evaluate the neutralization ability of the same antibody. The purified antibodies were diluted in a 3-fold gradient and mixed with DENV. The subsequent operation was consistent with the rapid screening of neutralizing antibodies described in Section 3.3. We used 3.125 µL of the FRNT_50_ for DENV I (corresponding to approximately 560 pfu), 25 µL for DENV II (corresponding to approximately 725 pfu), 12.5 µL for DENV III (corresponding to approximately 288 pfu), and 25 µL for DENV IV (corresponding to approximately 425 pfu). The PRNT for antibody 2E4-3G was performed simultaneously as a control.

**Figure 5:**
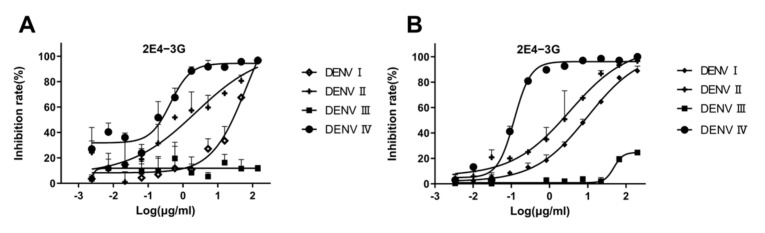
Validation of antibody neutralization activity. 2E4-3G is an antibody with a neutralizing activity obtained by the fluorescence reduction assay discussed in Section 3.3. (**A**) Antibody neutralization result obtained by an FRNT_50_ assay. (**B**) Antibody neutralization result obtained by a PRNT_50_ assay. Data are expressed as multipoint average relative fluorescence intensity (535 nm)/number of plaques ± standard deviation (SD) for two parallel wells in a 96-well/6-well plate. The FRNT_50_ and PRNT_50_ values were obtained via nonlinear regression by GraphPad Prism software. (FRNT, fluorescence reduction neutralization test; PRNT, plaque reduction neutralization test).

The neutralization curve of antibody 2E4-3G is shown in Figure 5A. When the antibody concentration is high, the inhibition rate of the virus is 100%. As 2E4-3G is diluted, the neutralization effect weakens until the inhibition rate becomes zero. The neutralization curve of 2E4-3G obtained from the FRNT is consistent with that of the PRNT (Figure 5B). Hence, it suggested that the newly designed FRNT can quantify the neutralization ability of the antibody. The most effective half-concentrations are shown in Table 3. Compared to the conventional PRNT_50_, the value of the FRNT_50_ was slightly elevated, probably owing to the relatively high dosage of the virus utilized. However, this was inevitable as a significant disparity from the blank well was necessary. Overall, the values of the FRNT_50_ and PRNT_50_ were basically consistent and remained within an order of magnitude of each other, which was a valid parameter to indicate the neutralization capacity of antibodies.

## 4. Discussion

In 2023, over 5 million dengue cases were confirmed globally, and more than 5000 dengue-related fatalities recorded [17,18]. Since the beginning of 2024, outbreaks of dengue fever have been reported in several countries, notably Brazil, Peru, and Argentina [3]. Consequently, the development of vaccines and antibodies against DENV has garnered significant attention. In drug development, a robust evaluation method can substantially accelerate the pace of drug discovery. In this study, various experimental vessels, incubation times, and culture medium components were employed to explore the optimal experimental system. Through comparative analysis under different conditions, the most widely applicable experimental system was identified: 48 h of incubation, a culture medium containing 2% FBS, and Thermo 137101. All four serotypes of dengue virus demonstrated favorable growth trends in this system. Utilizing this method, the preliminary screening of neutralizing activity for multiple antibodies was completed within three days; a process that typically required one week using conventional methods. Subsequently, the neutralizing activity of the selected antibodies was evaluated using this method and compared with traditional approaches. The results indicated that the outcomes of the FRNT_50_ were largely consistent with those of the PRNT_50_, which indicates that the sensitivity and specificity of the FRNT are highly comparable to those of the PRNT. In summary, the new FRNT can provide significant support for the development of anti-dengue virus antibody drugs.

Previously, the PRNT has traditionally been regarded as the gold standard for assessing antibodies against DENV–neutralization capacity, despite being a labor-intensive and time-consuming method [10,12,19]. Consequently, numerous simplified PRNTs have been developed for evaluating neutralizing antibodies against DENV [11,13,20,21,22]. However, these methods generally rely on manual counting of plaques or foci, which compromises their objectivity. Moreover, they are not suitable for evaluating multiple samples simultaneously due to the increased workload and time required. In contrast, we obtained FRNT results through multipoint scanning of 96-well plates under a wavelength of 535 nm, ensuring data objectivity compared to plaque-based methods. The new FRNT method, utilizing 96-well plates, allows for processing of a larger number of drug candidates within the same timeframe compared to the traditional methods using 6-well or 12-well plates. In addition, the new FRNT can be completed in just 48 h, significantly reducing the experimental duration compared to plaque-based methods that require 4–5 days. Most importantly, the new FRNT could evaluate the neutralizing activity of the antibodies. The FRNT_50_, essentially comprising the gold standard PRNT_50_, provides strong support for the development of neutralizing antibodies against DENV. Nonetheless, the new FRNT has certain limitations, such as the requirement for more viruses and the need to prepare the corresponding detection of antibodies, which could be optimized in future iterations. It is important to highlight that the commercial antibody 4G2 used in this study serves as a universal detection antibody for flaviviruses. This characteristic implies that the methodology may also be applicable to the development of antibodies or vaccines targeting other flaviviruses such as ZIKV, WNV, or JEV. However, due to limitations in experimental samples, validation for these additional applications was not conducted in this study. Should experimental conditions permit in the future, we plan to verify the broad-spectrum applicability of this method to other flaviviruses. In summary, this study has developed a novel screening and evaluation method, termed the FRNT, for anti-DENV neutralizing antibodies. Compared to conventional methods, the new FRNT offers enhanced speed, efficiency, and objectivity. We believe that the innovative FRNT could provide substantial support for the development of anti-DENV vaccines and antibodies, with potential applications extending to other flaviviruses.

## Figures and Tables

**Table 1:** Virus titer analysis.

Virus	Titer (pfu/mL)
DENV I	1.8 × 10^5^
DENV II	2.6 × 10^4^
DENV III	4.2 × 10^4^
DENV IV	1.7 × 10^4^

Calculation of dengue virus titer by plaque reduction test for dengue virus.

**Table 2:** Exploration and optimization of conditions for dengue virus (DENV) design of experiments.

Factor	Conditions
Incubation time	24 h/48 h/72 h
FBS concentration	0%/2%
96-well plate	Corning 3603/Thermo 137101
Cell seeding density	4 × 10^4^
4G2	1 μg/mL
Secondary antibody	1:3000

The conditions used in the viral titer assay—fluorescence test; if there is more than one condition, the one marked in red is the optimal condition.

**Table 3:** Antibody neutralization activity analysis.

Virus	FRNT_50_ (µg/mL)	PRNT_50_ (µg/mL)
DENV I	33.66	9.95
DENV II	2.33	3.38
DENV III	none	none
DENV IV	0.44	0.12

A comparison of antibody neutralization activity obtained using different methods. The FRNT_50_ and PRNT_50_ values were obtained via nonlinear regression by Graphpad Prism software 8.0.

## Data Availability

The data supporting the findings of this study can be obtained from the corresponding authors upon reasonable request.
